# Chiropractic manipulation in Adolescent Idiopathic Scoliosis: a pilot study

**DOI:** 10.1186/1746-1340-14-15

**Published:** 2006-08-21

**Authors:** Dale E Rowe, Ronald J Feise, Edward R Crowther, Jaroslaw P Grod, J Michael Menke, Charles H Goldsmith, Michael R Stoline, Thomas A Souza, Brandon Kambach

**Affiliations:** 1 Kalamazoo Center of Medical Studies, Michigan State University, 1000 Oakland Drive, Kalamazoo, Michigan, USA; 2 Institute of Evidence-Based Chiropractic, 6252 Rookery Road, Fort Collins, Colorado, USA; 3 Canadian Memorial Chiropractic College, 6100 Leslie Street, Toronto, Ontario, USA; 4 Program in Integrative Medicine, University of Arizona, 1503 East University Boulevard, Tucson, Arizona, USA; 5 McMaster University, 1280 Main Street West, Hamilton, Ontario, USA; 6 Western Michigan University, 1903 West Michigan Avenue, Kalamazoo, Michigan, USA; 7 Palmer-West College of Chiropractic, 90 East Tasman Drive, San Jose, California, USA

## Abstract

**Background:**

Adolescent idiopathic scoliosis (AIS) remains the most common deforming orthopedic condition in children. Increasingly, both adults and children are seeking complementary and alternative therapy, including chiropractic treatment, for a wide variety of health concerns. The scientific evidence supporting the use chiropractic intervention is inadequate. The purpose of this study was to conduct a pilot study and explore issues of safety, patient recruitment and compliance, treatment standardization, sham treatment refinement, inter-professional cooperation, quality assurance, and outcome measure selection.

**Methods:**

Six patients participated in this 6-month study, 5 of whom were female. One female was braced. The mean age of these patients was 14 years, and the mean Cobb angle was 22.2 degrees. The study design was a randomized controlled clinical trial with two independent and blinded observers. Three patients were treated by standard medical care (observation or brace treatment), two were treated with standard medical care plus chiropractic manipulation, and one was treated with standard medical care plus sham manipulation. The primary outcome measure was Cobb, and the psychosocial measure was Scoliosis Quality of Life Index.

**Results:**

Orthopedic surgeons and chiropractors were easily recruited and worked cooperatively throughout the trial. Patient recruitment and compliance was good. Chiropractic treatments were safely employed, and research protocols were successful.

**Conclusion:**

Overall, our pilot study showed the viability for a larger randomized trial. This pilot confirms the strength of existing protocols with amendments for use in a full randomized controlled trial.

**Trial registration:**

This trial has been assigned an international standard randomized controlled trial number by Current Controlled Trials, Ltd. . The number is ISRCTN41221647.

## Background

Adolescent idiopathic scoliosis (AIS) remains the most common deforming orthopedic condition in children [1,2]. It is manifested as a spinal curvature presenting at or about puberty which in its most aggressive form leads to progressive spinal curvature and vertebral rotation. In children it is associated with increased pain, reduced function and poor self-image [3,4]. In adults it is associated with increased back pain, poor quality of life and self-reported disability [5]. Estimates of prevalence of mild curvature, those varying from five to twenty degrees, is thought to be between 1 to 3% of adolescents with a male to female ratio of 1:2 [6,7]. In moderate to severe curves requiring medical management, the prevalence varies between .03 and .09% [7-11], with a male to female ratio of 1:7 [10,12].

Usual and customary medical management includes observation in early stages, bracing between 20 to 40 degrees curve progression, and surgical intervention for curves greater than 40 degrees [13]. Although bracing and surgery provide benefit, neither is free of shortfalls [14-16]. Bracing effectiveness is compromised by poor compliance [16], and surgical intervention is associated with negative side-effects (implant failure, wound infections and increased morbidity) [14,15]. These findings (both benefit and side-effects) are mostly based upon observational designed studies without controls [14-16].

Increasingly, adults [17,18] and children [19] are seeking complementary and alternative therapy, including chiropractic treatment, for a wide variety of health concerns. Approximately 2.7 million patient visits are made to American chiropractors each year for scoliosis and scoliosis-related complaints [20]. Chiropractors are using manipulation and other chiropractic approaches with these patients based largely on historical and anecdotal information, and without any scientific evidence.

A large scale, multi-disciplinary, collaborative clinical trial is needed to explore the effectiveness of chiropractic manipulation of patients with AIS. But prior to conducting such a trial, we needed to perform a pilot randomized controlled trial to explore issues of safety, patient recruitment and compliance, treatment standardization, sham treatment refinement, inter-professional cooperation, quality assurance, outcome measure selection and statistical analysis.

## Methods

### Design

The study design is a randomized controlled clinical trial with two independent and blinded observers. Two strata were studied: an unbraced group and a braced group. Each stratum had patients treated by standard medical care (observation or brace treatment), standard medical care plus chiropractic manipulation, or standard medical care plus sham manipulation. Patient blinding was attempted in the manipulation and sham manipulation interventions only.

### Sample specification

The target population was children aged 10–16 years who had been diagnosed (via x-ray) with AIS curves ranging from 20 to 30 degrees, and those with curves varying from 30 to 40 degrees who were undergoing bracing treatment. Study participants were recruited from the pediatric orthopedic clinic at The Kalamazoo Center for Medical Studies. Potential patients were screened using patient clinic records for inclusion and exclusion criteria. Those that met the study's criteria were invited to participate.

### Inclusion criteria

• Children aged 10–16 years

• Diagnosis of Adolescent Idiopathic Scoliosis

• Spinal curvature on P/A radiographs of between 20 and 30 degrees measured by the Cobb method in non-braced individuals and 30 to 40 degrees in braced individuals

• Palpatory evidence of subluxation (manipulative lesion) on chiropractic screening examination

• Signed informed consent by parent/guardian

• Signed child assent form

• Availability for follow-up evaluation

### Exclusion criteria

• Age <10 or >16 years

• Diagnosis other than AIS following clinical, radiographic and advanced imaging assessment

• Contraindications to manipulation: inflammatory arthritides, osteomyelitis, neoplasm, metabolic disturbances affecting the integrity of bone structure, fracture/dislocation/spinal instability, blood clotting disorders and connective tissue disorders

• Congenital or acquired structural spinal abnormalities

• Leg length inequality >3/8 inch (measured via x-ray)

• Pregnancy

• Pain as a primary clinical feature

• Mental incapacitation

• Previous back surgery

• Significant recent trauma

• Obesity impairing ability to manipulate

The Institutional Review Board of Borgess Medical Center, Kalamazoo approved the study protocol. Patients and their parents/guardians were informed and signed consent forms (parents/guardians) and assent forms (study participants) prior to participation. Following consent, further screening procedures and baseline data were gathered, including health history, physical exam (vital signs), neurological exam (deep tendon reflex, sensory deficit testing, muscle strength testing, Babinski), chiropractic spinal exam (static palpation, motion palpation, postural assessment), biomechanical evaluation (Shobers' modified-modified technique, Adams test, thoracic range of motion), quality-of-life self-report (Scoliosis Quality of Life Index, general health) and plain film radiographs. Patient recruitment occurred between March and July of 2003.

"Costs per randomization" were calculated by dividing the recruitment costs (the personnel time necessary to screen and recruit potential patients) by the number of randomizations. This information allowed assessment of future full-scale trial recruitment costs.

### Allocation

Prior to the start of the study, a computer-generated randomization schedule was prepared. Eligible patients were randomly assigned to 1 of 3 treatment groups (standard medical care, standard medical care plus chiropractic manipulation, or standard medical care plus sham manipulation). The allocation ratio was 1:1:1. Neither the participants nor the investigators knew whether a particular participant had been assigned to a study group or to a control group (standard medical care or standard medical care plus sham manipulation) until after assignment. The clinical research assistant e-mailed a member of the randomization team (off site) regarding the need for an assignment for a "qualified" participant by providing the clinic numeric code and bracing status. A member of the randomization team then provided the random assignment by return e-mail to the clinical research assistant.

Study participants randomized to the simulated and real treatment groups were given their choice of participating chiropractic treatment clinics. Once a clinic had been selected for attendance and treatment, the subject remained with that clinic for the duration of the trial. The treating chiropractor was provided with radiographs and Cobb angle measures for each study participant.

### Study sites

Participating treating chiropractors were recruited from Kalamazoo and Battle Creek, Michigan. Five chiropractic treatment centers were credentialed for the study and all used Diversified Technique as their primary method of treatment. Diversified technique is a widely used chiropractic manipulative technique that entails a high velocity, low-amplitude thrust.

Treating chiropractors were trained, tested and certified in study protocols and treatment methods. Three training sessions (approximately 2 hours each) were held at the medical center with the treating chiropractors, orthopedic surgeons and research staff. Each treating chiropractor was provided with a packet of training materials prior to the sessions. The first session focused on the study's research design and protocols and presented background information on adolescent idiopathic scoliosis. The second training session included an explanation of a standardized approach for detecting dysfunctional vertebrae, adjusting procedures and sham adjusting procedures. The final training session included a review of the study research design and protocols, standardized approach for detecting dysfunctional vertebras, adjusting procedures and sham adjusting techniques. During the last hour of the session, participants were evaluated using the Objective Structured Clinical Examination [21,22].

Upon entry to a participating chiropractic clinic, each subject underwent a standardized consultation, history and physical examination, including a chiropractic assessment. The patient and his or her parent/guardian were given a report of findings, a standardized explanation of chiropractic principles related to scoliosis, and an outline of the treatment schedule and procedures to be performed. On subsequent visits, pre-treatment analysis, treatment, post-treatment analysis and adverse reaction were recorded on standardized study treatment sheets. The treating chiropractors followed a scripted interview for each patient visit.

### Interventions

Usual and customary medical care for AIS patients with a Cobb of 20 to 25 degrees consists of careful observation (e.g., physical and radiographic examination twice a year). Patients with curves between 26 and 40 degrees are potential candidates for bracing with physical and radiographic examination twice a year, and patients beyond 40 degrees are potential candidates for surgery [23]. All groups received usual and customary medical care, including follow-up examination and x-ray at 6 months.

Active chiropractic treatment for this study consisted of prone, side posture and supine adjustments in conjunction with manual soft tissue therapy to the overlying tissues. Treatment consisted of the full spine chiropractic manipulation technique known as The Diversified Technique [24,25]. Because this was intended to be a pragmatic study within the domains of adjustive and soft tissue therapies, the specifics of the treatment (i.e. vertebral segments, direction of manipulation and use of soft tissue therapy) were left to the discretion of the treating practitioner.

Sham (pretend) chiropractic treatment consisted of a standardized approach which mimics regular chiropractic treatment, but which does not have the same mechanical effect. Subjects were placed prone on the treatment table and the spine was palpated lightly in a posterior-anterior direction. In this prone position, the head was rotated first to the right, then to the left, and held for a few moments while the chiropractor palpated the ankles and feet (a distraction maneuver). Following this distraction, the patient was positioned in a side-lying posture, and positioned for a low back adjustment (superior leg and hip flexed) without joint slack taken up. This position was held for a few moments, and the chiropractor contacted the soft tissues overlying the gluteal region and administered a light (non-therapeutic) impulse. This was repeated on the opposite side. The patient was then placed prone on the table, and the chiropractor administered a light impulse bilaterally to the muscles overlying the scapulae. The subject was then positioned supine, and the neck was palpated gently. Following this, the head was rotated to the side and held for a few moments, followed by a light impulse on the cranium over the external occipital protuberance.

### Schedule of visits

The schedule of treatment represents the frequency and duration of treatment typically used by the chiropractic profession. To form an estimate for the frequency and duration of care, a pre-study survey was administered to a random sample of American chiropractors (90% response rate) assessing treatment dosage for chiropractic management of AIS [26]. Generally, treatment consisted of three treatments per week for the first month, two treatments per week for the second month, one treatment per week for the third and fourth months, followed by a maintenance program of two treatments per month for the fifth and sixth months. Our treatment protocol was chosen based on the results of the survey of the American chiropractors [26]. For this effectiveness study, treating practitioners were allowed to increase or decrease the frequency of care, depending on clinical presentation (i.e. pain, postural changes, and changes evident on assessment). Patient compliance was deemed adequate when a patient received between 80% and 120% of the above-described treatments.

### Data collection and statistical analysis

All patients followed the same algorithmic protocols for initial evaluation and follow-up review. We collected data on radiographs, demographics, clinical history and quality-of-life domains at entry. Radiographs and quality-of-life measures were evaluated at baseline and at 6 months.

Cobb is the primary outcome measure for this study and is the gold standard for the measurement of curve magnitude in scoliosis [27-29]. Endorsed by the Scoliosis Research Society, measurement of the Cobb angle on full spine serial radiographs is used to make clinical decisions regarding initiation, termination and success of treatment. The reliability of the Cobb angle measure has been evaluated in many studies. When strict measurement protocols are used, consisting of sharpening marking instruments, standardizing protractors, and standardizing end plate selection, examiner error can be minimized [30]. In this study, strict measurement protocols were used and intra- and inter-examiner reliability was measured using two independent orthopedic surgeons blinded to treatment allocation.

Because there is a clinically important increase of curve severity (5 degrees) in moderate idiopathic scoliosis between morning and evening, the comparative x-rays were taken at approximately the same time of day (+one hour) as the entry x-ray [31]. Additionally, braced patients were required to remove their brace 6 hours prior to radiographic examination [32].

Quality of life is important in AIS because of the psychosocial stresses experienced by these patients. As a secondary outcome measure, we used Scoliosis Quality of Life Index (SQLI). This measure is a 22 item self-reporting health-related quality-of-life questionnaire for patients 10 to 18 years of age with idiopathic scoliosis [33]. SQLI is reliable (test-retest ICC 2,1; 0.80), valid (construct validity with Quality of Life Profile for Spine Deformities, Spearman's rho; 0.79) and demonstrates satisfactory distribution of scores. SQLI has five domains: physical activity performance (the presence and extent of physical limitations); back pain (the intensity and frequency of back pain); self-esteem (social confidence, self-regard, self-appearance, overall life assessment); moods and feelings (anxiety, depression and positive affect); and satisfaction with management. All scales are scored from zero (most pain, worst function, etc.) to 100 (no pain, best function, etc.). A global scale for SQLI (scored 0 to 100) was calculated by averaging the scores of all the scales.

Patient expectations about the therapeutic benefit of the treatment were assessed before randomization [34]. Allowing only naive patients is problematic, because it may be difficult to recruit this population. Furthermore, an expectation for or against chiropractic could influence patient scores on subjective measures. If patient preference is disproportionately distributed among the groups, there may be an inflated threat of obtaining a skewed outcome [35]. Patients for this study were asked to describe their expectations for improvement of their spinal condition (without regard to treatment) using a 5-point scale with choices varying from "very much improved," to "very much worsened." The patients then rated how helpful they believed chiropractic would be for their current spinal problems using a 5-point scale with choices varying from "very helpful" to "very unhelpful".

To provide proof of blinding for patients in the real and sham manipulation groups, patients were questioned after all therapy had been administered about whether they received active or inactive therapy. A 7-point scale was used with choices varying from "definitely real therapy," to "definitely pretend therapy." We also asked patients about co-interventions and contamination during the study period.

Touch screen technology (TST) was used to administer SQLI and expectation, blinding, co-Intervention and contamination questions. The quality of the data collected with the touch-screen system has been reported as good, with no missed responses [36,37]. This method eliminates possible entry errors and the need for double-entry checks. Additionally, it is well accepted by patients, the majority of whom find all aspects of the TST system easy to use [38].

Quality assurance procedures were established and implemented for all aspects of the trial. We developed a manual of operational procedures which included operational definitions of recruitment, measurement procedures, etc. All forms were standardized (pre-coded, self explanatory, easy to read, coherent, pretested, and labeled on every page with an ID number).

Simple descriptive analyses were used to report the findings. Outcome measures were not aggregated, but were reported individually because of insufficient sample size and the high risk of committing sampling errors. To assess intra- and inter-reliability, intraclass correlation coefficients (ICC 2,1) were calculated for the Cobb measures [39]. All data were entered into a spreadsheet and analyzed with Minitab 12 (State College, PA). All data were checked for accuracy.

## Results

Baseline characteristics of the subjects are presented in table 1. These patients reported no comorbidities, recent accidental injuries, previous chiropractic treatment or significant pain associated with their scoliosis. Self report of general health using a question from the SF-36 scale was rated as either "very good" or "good" for these participants, and all thought chiropractic treatment could be somewhat helpful to their condition. Patients' expectations for future improvement of their spinal condition was rated as "very much improved" by one patient, "somewhat improved" by two patients, and "neither" by three patients.

**Table 1:** Patient characteristics at baseline.

**Group, patient #**	**Gender**	**Age (years)**	**Race**	**Family history**	**Wt. (lb.)**	**Ht. (inches)**	**Menses**	**Braced**
medical-1	female	13	c	no	112	62	yes	no
medical-2	male	16	c	yes	159	75	-	no
medical-3	female	10	c	no	67	54	no	yes
sham	female	16	c	yes	113	65	yes	no
chiropractic-1	female	16	aa	yes	109	66	yes	no
chiropractic-2	female	13	c	no	75	62	no	no

aa = African American, c = Caucasian; Family History = family history of scoliosis, medical = standard medical care, sham = standard medical care plus sham manipulation, chiropractic = standard medical care plus chiropractic manipulation

Table 2 describes AIS radiograph assessment categories. Riser sign describes bone maturity (0 = immature to 5 = mature) [40], and Nash-Moe classification describes vertebral body rotation (0 is not rotated, 3 is fifty percent rotated) [41]. The Lenke classification system consists of 3 components: Lenke curve type (the curve types have specific characteristics that differentiate structural and nonstructural curves in the proximal thoracic, main thoracic, and thoracolumbar-lumbar regions), the lumbar spine modifier (based on the relation of the center sacral vertical line to the apex of the lumbar curve) and the sagital thoracic modifier (differentiates the degree of Cobb Angle; T5–T12 Cobb Angle from 10 to 40 degrees is N and above is "+") [42]. Inter-rater reliability, estimated as percent of agreement for the scoliosis classifications (Risser, Nash-Moe, Lenke curve type, Lenke spine modifier, and Lenke sagittal thoracics modifier), varied between 0.72 and 0.92.

**Table 2:** Patient radiographic variables at baseline.

**Group, Patient #**	**Risser sign**	**Nash-Moe apical vertebrae**	**Lenke curve type**	**Lenke spine modifier**	**Lenke sagittal thoracics modifier**
medical-1	3	2	2	A	+
medical-2	4	2	1	A	N
medical-3	0	1	5	C	N
sham	4	1	3	B	N
chiropractic-1	4	2	1	A	N
chiropractic-2	1	2	5	C	N

medical = standard medical care, sham = standard medical care plus sham manipulation, chiropractic = standard medical care plus chiropractic manipulation

Table 3 demonstrates the findings of the Cobb Angle pre- and post-testing. Every patient had more than one curve that was measured at baseline and at follow-up. The intra-examiner reliability intraclass correlation coefficients (ICC 2,1) for Cobb measurement scores were 0.96 and 0.98 with inter-examiner at 0.96. (SEM 0.69 and SD 1.38). Although, there is no gold standard for declaring the failure or success of progression for Cobb, Rowe recommended 10 degrees from start of treatment, and Nachemson used 6 degrees or more on two consecutive radiographs [16,32]. We tested two discriminatory points by establishing benchmarks at 6 degrees and 10 degrees. Failure was deemed as a progression of at least 6 degrees or at least 10 degrees, and success was deemed as curve improvement of at least 6 degrees or at least 10 degrees. For the 6 degree benchmark, the standard medical care group had no curves that achieved success and one curve that was rated as a failure; the standard medical care plus sham manipulation patient had both curves rated as failures; and the standard medical care plus chiropractic manipulation group had no curves that achieved failure and one curve that was rated as a success. For the 10 degree benchmark, the standard medical care group had no curves that achieved success or failure; the standard medical care plus sham manipulation patient had one curve rated as failure; and the standard medical care plus chiropractic manipulation group had no curves that achieved failure and one curve that was rated as a success. Chiropractic manipulation was delivered on 52 visits and resulted in two benign reactions. One reaction with a moderate amount of pain lasted for 24 hours; the other produced mild pain lasting 6 hours. Neither reaction reduced the normal activities of the patients. All patients meet the established compliance standards. The patient in the sham group thought she probably had real treatment. Whereas, the chiropractic treatment patients thought their treatment was definitely real or possibly real.

**Table 3:** Primary Outcome Measure: Cobb Angle (n = 6*).

**Group, patient #**	**Curve pattern**	**Baseline**	**Follow-up**	**Pre-Post Difference**	**Success @ 6°**	**Failure @ 6°**	**Success @ 10°**	**Failure @ 10°**
medical-1	Thoracic	28	28	0				
medical-1	Thoracic	39	42	+3				
medical-1	Thoracic-Lumbar	20	22	+2				
medical-2	Thoracic	22	22	0				
medical-2	Thoracic	27	33	+6		**x**		
medical-3	Thoracic	13	13	0				
medical-3	Thoracic-Lumbar	22	26	+4				
sham	Thoracic	10	22	+12		**x**		**x**
sham	Lumbar	18	24	+6		**x**		
chiropractic-1	Thoracic-Lumbar	26	22	-4				
chiropractic-1	Lumbar	17	14	-3				
chiropractic-2	Thoracic	18	18	0				
chiropractic-2	Lumbar	29	18	-11	**x**		**x**	

* Patients had more than one curve measured at baseline and at follow-up.

medical = standard medical care, sham = standard medical care plus sham manipulation, chiropractic = standard medical care plus chiropractic manipulation

Table 4 documents the findings from measuring quality of life domains before and after the intervention period. An absolute change in score of more than 15 points is considered minimum clinically important for either the sub-scales or global score [33]. The standard medical care group had one patient whose scores in the domains of self-esteem, back pain, physical activity, moods and feelings and the global score expressed a clinically important deterioration. This patient was compliant with the study protocols, including follow-up x-ray, but underwent surgical therapy. The quality of life measure was not administered until after surgery The standard medical care plus sham manipulation patient expressed no clinically important changes from baseline. The standard medical care plus chiropractic manipulation group had one patient who reported a clinically important improvement in the moods and feelings domain and the global score.

**Table 4:** Scoliosis Quality of Life Index pre – post.

**Group, patient #**	**PRE self-esteem**	**POST self-esteem**	**PRE back pain**	**POST back pain**	**PRE physical activity**	**POST physical activity**	**PRE moods feelings**	**POST moods feelings**	**PRE global**	**POST global**	**POST therapy satisfaction**
medical-1	70		90		85		85		82.5		
medical-2	80	80	95	100	90	90	70	85	83.75	88.75	
medical-3	75	70	100	100	90	90	85	100	87.5	90	87.5
sham	80	95	100	100	90	90	100	100	92.5	96.25	100
chiro-1	95	100	95	95	85	85	40	100	78.75	95	87.5
chiro-2	80	90	100	100	90	90	100	100	92.5	95	87.5

All scales are scored from zero (most pain, worst function, etc.) to 100 (no pain, best function, etc.); medical = standard medical care; sham = standard medical care plus sham manipulation; chiropractic = standard medical care plus chiropractic manipulation. **Note: **The post-test for medical patient number one (medical-1) occurred after spinal surgery and is not applicable to this study; medical patient number two (medical-2) was not asked the satisfaction question, because no "active" therapy was applied; and medical patient number three (medical-3) was braced.

### Health provider recruitment

We easily recruited both orthopedic surgeons and chiropractors for this study. Treating chiropractors were in practice a mean of 11.6 years (min. 2.5 years to max. 30 years). In an exit survey, orthopedic surgeons and chiropractors rated cooperation between groups above 9, with 10 representing "extremely cooperative" and 0 "not cooperative." Treating chiropractors rated our training program at 9, with 10 representing "extremely effective" and 0 "not effective." The treating chiropractors felt no burden to their practice during this study, and all would participate in a full study. The treating chiropractors, orthopedic surgeons and research team rated the overall experience of the study at 8, with 10 representing an "outstanding experience" and 0 a "horrible experience."

### Protocol compliance

Observation by the research team found no breach in standardized chiropractic treatment. An exit survey of patients, treating chiropractors, research staff and orthopedic surgeons found one patient with a co-intervention. That patient, from the standard medical care group, underwent a surgical intervention. This study suffered from one protocol failure: the sham patient received 34 visits (8 visits more than the ideal of 26 visits), but this did not exceed our compliance upper limit of 38 visits. Our exit meeting of treating chiropractors, orthopedic surgeons and research team produced strategies to improve the protocols, data collection, patient recruitment and chiropractic practitioner training process for the full study.

### Patient recruitment

**Figure 1:**
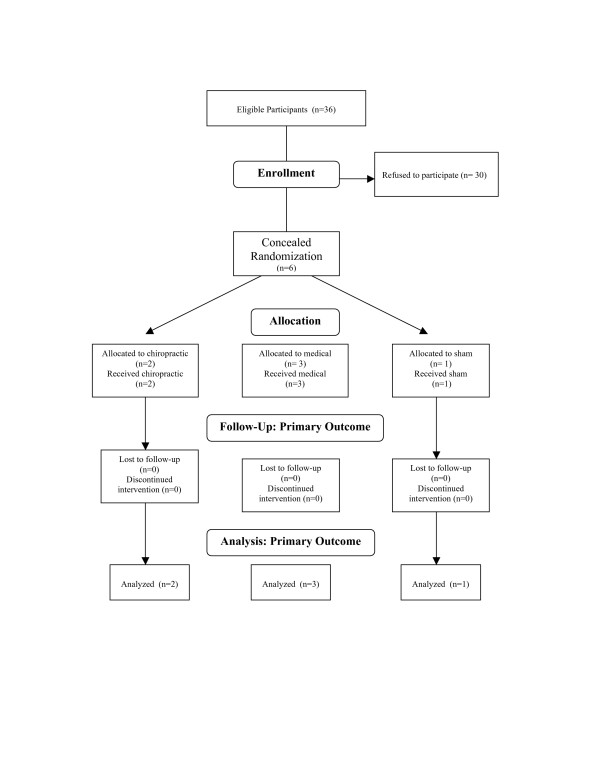
Flow of Participant.

This study had an enrolment rate of 17% (36 patients were eligible, and 6 patients were randomized). Of the 30 patients who qualified but refused to participate, 12 said they did not have enough time, 8 gave no reason, and the remainder had a variety of reasons (transportation, not interested in participating, family issues). All randomized patients accepted assignment (figure 1). Costs for randomization were calculated at $360/randomization (the total cost of personnel time necessary to screen and recruit potential patients was $2,176; US Dollars 2003).

## Discussion

Overall, our pilot study showed the viability for a larger randomized trial. Orthopedic surgeons and chiropractors were easily recruited and worked cooperatively throughout the trial. Patient recruitment and compliance was good. Chiropractic treatments were safely employed, and research protocols were successful.

A vital component of conducting a trial is the ability to recruit eligible participants. The recruitment rate for this study was 17%. It is important to note that this rate is comparable to the rates of several other chiropractic trials which had a mean recruitment rate of 17.2%; min to max 6 to 37% [43-47]. Costs for randomization were calculated at $360/randomization. This is well below the cost of other studies, where costs were over $900/randomization [45].

Forty percent of the potential participants declined involvement in the trial, because they felt they did not have enough time. Our patient population, adolescents, are commonly known to have busy schedules, and it is speculated that potential participants and families did not want to invest 6 months of time with the possibility of receiving placebo treatment. Forthcoming recruitment efforts will need to weigh the advantages and disadvantages of a sham arm. We were successful in blinding the patient in the sham group to placebo chiropractic treatment, but patients in the real group guessed the validity of their care. Moreover, blinding is likely to be problematic in the full study [48]. This reduces the benefit of using a placebo group. Additionally, it is unlikely that AIS, as measured by Cobb, can be influenced by non-specific effects. Miller states that it may not be feasible to use a placebo arm in some complimentary and alternative therapies, such as in spinal manipulation [48]. In these cases, he recommends a no-treatment control and a priori requirement of a minimum clinically important target difference in the primary outcome measure.

All of the subjects completed the six-month treatment protocol and were compliant with treatment schedules. One patient, from the medical care group, required a surgical co-intervention. But all other patients avoided confounding treatments. The data collection process performed well, without missing data for important variables. The data collection forms captured baseline data, treatment visits and exit data and were received in a timely fashion from all study providers.

In spite of one protocol failure, study participants and treating practitioners were able to maintain compliance with standard procedures. In part, the success of adherence to study protocols was due to the training program. In an exit survey with 100% response rate, treating chiropractors rated our training program as very good. In post study meetings with treating chiropractors, orthopedic surgeons and research investigators, strategies to improve future protocols were discussed. These strategies included the following: 1) The need for more chiropractic treatment centers located near potential patients, so we can offer a wider geographic catchment; 2) The need to provide our treating chiropractors with a brief review of the study protocols immediately before practitioners meet their first research patient, so the protocols are easier for our treating practitioners to use with a first patient; 3) The need to modify our inclusion criteria to accept patients aged 8 to 14 years with a Risser of 0 or 1, so that our group is more likely to be homogenous and to have a progression of the curves; 4) The need to modify the treatment schedule to 3 months of active treatment with once-a-month follow-ups, so we can improve patient recruitment; 5) The need to measure both cost-effectiveness and brace compliance; 6) The need to use a follow-up period of at least four years [32].

The development, implementation and successful completion of a large randomized controlled trial would necessitate cooperation among chiropractors and orthopedic specialists, culturally divergent health professionals. In this study, both orthopedic surgeons and chiropractors were easily recruited for participation. In an exit survey, both groups rated inter-professional relationship as extremely cooperative, and their overall experience of the study was very positive. No counter-productive events occurred. Overall, the treating chiropractors and orthopedic surgeons felt little burden to their practice during this study, and all would participate in a full study. It is evident that chiropractors and medical specialist can work together for the benefit of mutual patients.

Contrary to the concerns of some, few negative side effects were noted among study participants receiving real or sham interventions. No subjects withdrew or were removed from the study because of an adverse effect. Among those receiving chiropractic treatments, approximately 120 interventions were provided. Of these treatments, two resulted in benign reactions. Neither reaction negatively impacted normal activities of the subjects. For the study participants receiving the sham treatment, no negative reaction was noted. These outcomes are consistent with other studies employing spinal manipulative interventions in pediatric patients [49]. Recognizing that manipulative therapy applied to pediatric patients with scoliotic changes might increase risk of injury, we endeavoured to chart pain outcomes following treatment. Less than 1% of treatments provided resulted in any noted discomfort.

Caution is needed in the interpretation of data from any pilot study. No conclusion about the effectiveness can be rendered. It is impossible to make any causal inferences. The findings may be the result of non-treatment factors. Small sample sizes are known to have unequal distributions of important prognostic variables. A large sample of patients in a well-designed randomized clinical trial with an appropriate follow-up period must be examined before conclusions can be rendered.

## Conclusion

A pilot study is essential to the planning of a well-designed full-scale trial, because a number of important issues need to be unravelled before time and suitable funding is committed. The results of this pilot study suggest that it is feasible to recruit AIS patients for a randomized clinical trial to compare chiropractic care and standard medical care. This pilot also supports the utility of existing protocols, with necessary amendments, for a full randomized controlled trial.

## Competing interests

The author(s) declare that they have no competing interests.

## Authors' contributions

DER participated in design, acquisition of data, analysis and interpretation of data, drafting the article and revising the article critically for important intellectual content. RJF conceived of the study, participated in design, acquisition of data, analysis and interpretation of data, drafting the article and revising the article critically for important intellectual content. ERC participated in design, acquisition of data, drafting the article and revising the article critically for important intellectual content. JPG participated in design, acquisition of data, drafting the article and revising the article critically for important intellectual content. JMM participated in design, analysis and interpretation of data and revising the article critically for important intellectual content. CHG participated in design and revising the article critically for important intellectual content. MRS participated in design, analysis of data, and editing the statistical portion of the article. TAS participated in design and revising the article critically for important intellectual content. BK participated in acquisition of data. All authors read and approved the final manuscript.
